# European Public Health News

**DOI:** 10.1093/eurpub/ckac088

**Published:** 2022-08-01

**Authors:** Dineke Zeegers Paget, Dineke Zeegers Paget, Hedinn Halldorsson, Clayton Hamilton, Hans Henri P Kluge, Agnese Pastorino, Gerald Rockenschaub, Cristiana Salvi, Floris Barnhoorn

**Affiliations:** EUPHA Strategic advisor; EUPHA Strategic advisor; Deputy Director EPH Conference

This is the last European public health news that I am organizing. In my contribution, I reflect on the past as well as welcome my excellent successor, Dr Marieke Verschuuren. Halldorsson et al. address the problem of infodemics and WHO Regional Office for Europe is urging national governments to strengthen risk communication and community engagement. They conclude that reinforcing public health responses to infodemics and improving preparedness for future crises are the role of everyone. Barnhoorn addresses the topic of the European Health Data Space (EHDS), a topic that is well represented in the conference in Berlin 2022. The EHDS is a new framework to make it easier to access and exchange information about the health of European citizens, thereby obviously taking into account the EU’s high protection standards.

## A breath of fresh air at EUPHA office

Zeegers PagetDinekeEUPHA Strategic advisor

On the 1 July 2022, I am stepping down as director of EUPHA. After 25 years of being EUPHA director, it is time for a breath of fresh air at EUPHA office. My successor, Dr Marieke Verschuuren, is ready to give the office and our activities a new push in the right direction.

Over the years, I have worked for EUPHA in different places. I started in my office/bedroom in Bern, Switzerland, with 1 day a week working for EUPHA. Quite a challenge to work with three small children in the house. But it was certainly a very good way to slowly get more and more involved in EUPHA’s work. In 2000, the whole family packed up to move to the Netherlands and the work of EUPHA slowly increased, first to 2 days a week at the Netherlands School of Public Health and then to a full-time position at NIVEL, where we have been since 2002. In the beginning there was just me, but the team grew larger and larger and we now have five expert staff members in the Netherlands and one in our Brussels office. We still receive support from NIVEL in the person of Ingeborg, our financial officer, and we are very grateful to have her on the team.

The first conference I attended was the one in Pamplona in 1997. In those days, the conferences were still organized at universities using the smaller teaching rooms as break-out rooms. The plenary programme was dedicated to research and several excellent researchers presented their work. In Pamplona, there was a mix of Spanish and English sessions, a programme only repeated in 2000 in Paris with French and English sessions, due to a French law insisting that any conference organized in France should be in French first. Over the years, the conferences grew in size and in quality. The abstract submissions never stop to amaze me with a steady increase in single as well as workshop submissions. It makes the programme planning all the more difficult, but the programme broader and more interesting. This year, Berlin 2022, had so many high-quality workshop submissions that it was decided to invite some of the rejected workshops to be organized as webinars—a sort of appetizer for the Berlin 2022 conference. The plenary sessions have changed as well with more emphasis on policy and research to policy. There are now several partners (European Commission-DG Sante, ECDC, WHO regional office for Europe, EuroHealthNet, EHMA, ASPHER, etc.) organizing plenary sessions and being active in the parallel programme as well. It is no longer a EUPHA conference, it is (for 15 years now) the European Public Health Conference.

The EUPHA sections—theme-specific working groups—have made an enormous development. From four sections in 1997 to 26 sections in 2022. And these sections touch upon all main issues in public health. Their representation of EUPHA in meetings, advisory fora, discussions with DG Sante, etc. are exemplary and I am so proud of the work they are doing to increase the visibility of EUPHA. Every year, the Section Council meeting is organized in February to discuss joint activities and joint workshops. This meeting is like a family gathering and brings along so much positive energy that lasts at least until the conference in November. And every new section president, new section, new office member has been automatically integrated in this family.

In 2011, it was decided to set up EUPHAnxt, a network to help EUPHA invest in the younger generation. The idea came from three Italian interns at EUPHA office and EUPHAnxt has—since then—grown both in activities, subscribers and representation. It was clear that the main EUPHAnxt coordinator should have a seat in the Executive Council to make sure the young are heard. Over the years, EUPHAnxt has grown into a wonderful, well-connected network of public health students and young professionals and the added value to our network is amazing. I loved working with the coordinators of the network, they see the world of public health differently from someone who has been in the field for very long. Their new ideas included the EUPHAnxt fellows at the conference, pub quizzes to get to know each other and mentoring sessions with well-renowned public health experts.

Our EUPHA members have been a great asset to EUPHA. In the beginning, contact with the members was limited to once a year during the annual meeting, unless the member was co-organizing the conference. Over the years, I have enjoyed getting to know our members, learning from their experiences as well as supporting them whenever I could. EUPHA members have become more active, more visible and I highly appreciate that development. Working with individual member associations, for instance with the UK Faculty of Public Health organizing law and public health webinars or the Spanish society providing the template for our Rapid Responses Team, has been immensely rewarding. Our annual meeting has become the highlight of the year for me, seeing everybody, discussing the future of EUPHA and selecting members for the EUPHA Executive Council.

I have been privileged to work with the members of the EUPHA Executive Council, all with their own expertise, all dedicated to further EUPHA’s work. And there have been some big names in the Executive Council—Martin McKee, Peter Allebeck, Walter Ricciardi, Natasha Azzopardi Muscat and Iveta Nagyova to name but a few. I have learned so much from each of them and I treasure the hours of discussion mostly in agreement, sometimes in disagreement in this Council.

Over the years, I have seen my working hours increase from 4 h/week to 36 h/week then back to 32 h/week. In the last couple of years, I have been working 16–20 h/week taking into account the aftermath of my battle against cancer. I feel I do no longer have the energy to—together with the Executive Council—lead EUPHA to even more visibility, sustainability and success. EUPHA needed new energy at the office to lead the team, to take EUPHA further, to be the face of EUPHA and to ensure that EUPHA continues for another 30 years. In Marieke Verschuuren, EUPHA has found the right energetic person to achieve our future goals. I look forward to working with her as I am happy that I can remain involved in EUPHA as an advisor and I continue to be a EUPHA family member.

## WHO Regional Office for Europe: How to quell the next infodemic EJPH, August 2022

HalldorssonHedinnHamiltonClaytonKlugeHans Henri PPastorinoAgneseRockenschaubGeraldSalviCristiana

Although the term ‘*infodemic’* was first coined in 2003, it wasn’t widely understood until 17 years later. Today, as the world continues to respond to COVID-19, and health institutions struggle more than ever to reach audiences with scientifically sound information, no explanation of the term is needed.

In the 2021 report of the UN Secretary General, *Our Common Agenda,* the ability to undermine scientifically established facts is described as an existential risk to humanity. The lion’s share of today’s greatest global challenges requires collective decisions and action informed by the best available data. What blurs the picture, sabotages or misinterprets scientific evidence and makes it difficult to distinguish between right and wrong—is misinformation and disinformation. The first is factually incorrect information, while the latter is deliberately deceptive. Both, when widely available, are dangerous.

That’s because exposure to false information can kill—and has killed during the COVID-19 pandemic. The uncertainty that often reigns at the beginning of an emergency nourishes a lack of trust towards authorities and encourages the spread of false, excessive and misleading health information that undermines an effective response. In a broader context, infodemics fuel social instability. Beyond potential physical harm, there are psychological risks that can have adverse effects on people’s emotional well-being and mental health.

Easy access to information is beneficial when knowledge gaps are filled in a timely way with appropriate, actionable and scientifically sound messaging, but becomes a threat when incorrect, harmful messaging goes viral. Connectivity is therefore a double-edged sword—that the WHO European Region experienced when its 53 Member States faced a pandemic and an infodemic at the same time.

When analyzing the underlying factors behind the COVID-19 infodemic, they are not purely digital, but rooted in complex historical, sociocultural contexts. Among the main cognitive, behavioural contributors are cultural and religious beliefs, distrust towards health institutions and low levels of health literacy.

Several studies show how COVID-19 misinformation is one of the causes of delayed outbreak responses, due to the slowed adoption of preventive measures. Higher exposure to online misinformation is also associated with vaccine hesitancy, contributing to lower COVID-19 vaccination rates.

During the pandemic, we have seen higher levels of anxiety in people who believed in unfounded conspiracy theories—including those concerning biological warfare and 5G networks. The data also show a clear correlation between misinformation and symptoms of anxiety, depression and post-traumatic stress disorder.

We’ve seen misinformation diffused through social media and messaging applications increasing mistrust in health workers and institutions, even resulting in episodes of violence against medical staff. In some communities, distrust towards experts—fueled by the infodemic—led to self-medication, fear and misuse of pharmaceuticals. Often, users exposed to inaccurate information exhibited unhealthy and risky behaviour for themselves and their communities.

The societal havoc caused by the SARS-CoV-2 virus offers countless lessons on how to apply digital tools to prevent and mitigate infodemics. The World Health Organization is using this experience to curb the spread of misinformation and protect against it in the future. Our aim is to help the public make the right choices and assist national authorities to become ‘the trusted source’ of health information at national level.

Since social media and the Internet are important platforms for disseminating large amounts of false information, they are also key spaces where solutions are found and can be implemented. These solutions are not only digital, but also political, regulatory, educational as well as communications based. Key among them are

- fact-checking, false information reporting mechanisms;
- social listening tools augmented by artificial intelligence that help analyze large, fast-flowing data, assess risks and identify signals;
- monitoring programmes, multi-stakeholders’ coordination and regulatory frameworks that respect freedom of expression;
- digital health literacy that improves the ability to spot misinformation.

These solutions are truly effective when integrated with offline practices that engage communities to monitor and debunk false information through trusted actors and networks. As offline and online environments are intertwined, infodemic responses in both spheres need to complement one another.

WHO’s policy recommendations, applicable at a national, regional and global level, are clear. We urge national governments to strengthen risk communication and community engagement and, as part of that, reinforce multistakeholder networks for infodemic management. We call for continuous monitoring of online and offline content, focus on improving health literacy, infodemic management trainings and safe platforms where protection from harmful content is enhanced.

Reinforcing public health responses to infodemics and improving preparedness for future crises is not the role of a single actor. It is everyone’s role.

Success depends on a whole-of-society approach. National authorities, journalists, fact-checkers, the information technology sector, policy-makers, civil society, social media users and the public, all play a vital role in halting the spread of harmful information. Convening all these stakeholders under one umbrella, the WHO European Region is working towards the first European Infodemic Preparedness and Response Alliance (EIPRA). In line with our Digital Health Action Plan, WHO/Europe aims to boost the capacity to manage future infodemics in country-specific contexts and foster innovation on predictive analytics through big data and artificial intelligence.

The pandemic may well have ebbed in some countries in the WHO European Region, but the infodemic has not. Other pandemics will arrive and when they hit, they are likely to be accompanied by infodemics. Both claim lives. Both can be counteracted, *if* we implement the lessons we have learned from COVID-19.

WHO is developing the tools. It is then up to national governments and the public to make use of them. For everyone’s greater good.

Please find the policy brief *Digital solutions to health risks raised by the COVID-19 infodemic in the publications database of the WHO Regional Office for Europe:*https://www.who.int/europe/publications/i

https://apps.who.int/iris/handle/10665/356315

## 15th European Public Health Conference 9–12 November 2022 hub27 Berlin, Germany

BarnhoornFloris
Deputy Director EPH Conference

### EPH Conference programme pays attention to the future European Health Data Space

On 3 May, the European Commission published its plans for the EHDS, a new framework designed to make it easier for researchers, regulators and individuals to access and use information about the health of millions of citizens across the European Union. The network aims to create a genuine single market for electronic health record systems—a key pillar of the European Health Union—following the EU’s high data protection standards.

The objectives of the EHDS are

- Empower individuals through better digital access to their personal health data and support free movement by ensuring that health data follow people;
- Unleash the data economy by fostering a genuine single market for digital health services and products and
- Set up strict rules for the use of individual’s non-identifiable health data for research, innovation, policy-making and regulatory activities.

The EHDS plan covers the use of primary data, secondary data, common governance and synergies with other health policy priorities. In terms of primary data, the EHDS aims to enable the millions of citizens of the EU to access their own health data and make it available to a health professional of their choice, including when abroad and in other languages and also enable health professionals to update those records.

In terms of secondary use of data, the EHDS aims to set out a common EU framework allowing for use of anonymized health data of EU citizens for research, innovation in public health, policymaking, regulatory activities and personalized medicine. It will draw on the creation of a new and decentralized EU infrastructure for secondary use of health data called HealthData@EU which will connect access bodies in all EU member states.

Naturally, the conference programme pays extensive attention to the EHDS plan. First of all through a plenary session entitled *Benefits and challenges of the European Health Data Space*, organized by EUPHA and the European Commission and scheduled for Thursday 10 November, 17:40–18:40 CET. The plenary session will discuss not only the expected benefits from the EHDS, but also the challenges related to technology, governance and privacy. The exchange of data at European level means that health data from different sources need to be able to talk to each other. Making the data Findable, Accessible, Interoperable and Re-usable (FAIR) is key to the success of the EHDS. Moreover, the diversity of Europe’s health information systems needs to be taken in account. The EHDS will also have to be transparent to ensure privacy of personal information included in the EHDS. Speakers include representatives from the European Commission, health data regulators and research and patient’s organizations.

In addition to the plenary session, we offer two workshops in the EPH Conference parallel programme (dates/time to be announced later).

### The European Health Data Space 2: is Europe ready to maximise the re-use of health data?, organized by Sciensano, Belgium

The EU’s Heads of State called in October 2020 to set up a EHDS: ‘The European Council welcomes the creation of common European data spaces in strategic sectors, and in particular invites the Commission to give priority to the health data space’. As a response, multiple initiatives were set-up to engage the views and expectations from EU member states in the development of the upcoming legislative proposal on the EHDS for primary (EHDS1) and secondary use of health data (EHDS2). One of these was the launch of the Joint Action (JA) Towards the EHDS (TEHDAS) in February 2021.

### European Health Data Space: Dialogue on TEHDAS JA data quality and semantic interoperability proposals, organized by the Joint Action TEHDAS

Quality of digital health data has been the focus of research and debate for a long time and several proposals for improvement have been put forward. While these efforts are ongoing, secondary use combined with recent developments in data analytics, AI and real-world data, have raised new requirements on our understanding of data quality and the means for its assurance. Both the European Commission and Member States have underlined the need to support EU and national authorities, as well as the scientific community to agree on interoperability guidelines addressing data quality and semantic interoperability. The TEHDAS Joint Action has responded to these expectations. Key members of the TEHDAS team will present the process through which the TEHDAS JA recommendations on data quality and semantic interoperability have been developed, the results achieved thus far and the remaining open questions.

The EHDS is a topic that concerns all public health researchers and delegates at the EPH Conference and we expect a high turnout.

See you in Berlin.

